# Association of *HLA-DRB1* amino acid residues with giant cell arteritis: genetic association study, meta-analysis and geo-epidemiological investigation

**DOI:** 10.1186/s13075-015-0692-4

**Published:** 2015-07-30

**Authors:** Sarah Louise Mackie, John C. Taylor, Lubna Haroon-Rashid, Stephen Martin, Bhaskar Dasgupta, Andrew Gough, Michael Green, Lesley Hordon, Stephen Jarrett, Colin T. Pease, Jennifer H. Barrett, Richard Watts, Ann W. Morgan, UK GCA Consortium, UKRAG Consortium

**Affiliations:** School of Medicine and NIHR-Leeds Biomedical Research Unit, Chapel Allerton Hospital, Leeds, LS7 4SA West Yorkshire UK; Department of Rheumatology, Southend University Hospital, Prittlewell Chase, Southend, SS0 0RY Essex UK; Department of Rheumatology, Harrogate and District Foundation NHS Trust, Lancaster Park Road, Harrogate, HG2 7SX North Yorkshire UK; Department of Rheumatology, York Teaching Hospital NHS Foundation Trust, Wigginton Road, York, YO31 8HE North Yorkshire UK; Department of Rheumatology, Dewsbury and District Hospital, Halifax Road, Dewsbury, WF13 4HS West Yorkshire UK; Department of Rheumatology, Pinderfields General Hospital, Aberford Road, Wakefield, WF1 4DG West Yorkshire UK; Department of Rheumatology, Chapel Allerton Hospital, Leeds, Leeds, LS7 4SA West Yorkshire UK; Department of Rheumatology, Ipswich Hospital NHS Trust, Heath Road, Ipswich, IP4 5PD Suffolk UK; Wellcome Trust Brenner Building, St. James’s University Hospital, Beckett Street, Leeds, LS9 7TF West Yorkshire UK

## Abstract

**Introduction:**

Giant cell arteritis (GCA) is an autoimmune disease commonest in Northern Europe and Scandinavia. Previous studies report various associations with HLA-DRB1*04 and HLA-DRB1*01; *HLA-DRB1* alleles show a gradient in population prevalence within Europe. Our aims were (1) to determine which amino acid residues within *HLA-DRB1* best explained *HLA-DRB1* allele susceptibility and protective effects in GCA, seen in UK data combined in meta-analysis with previously published data, and (2) to determine whether the incidence of GCA in different countries is associated with the population prevalence of the *HLA-DRB1* alleles that we identified in our meta-analysis.

**Methods:**

GCA patients from the UK GCA Consortium were genotyped by using single-strand oligonucleotide polymerization, allele-specific polymerase chain reaction, and direct sequencing. Meta-analysis was used to compare and combine our results with published data, and public databases were used to identify amino acid residues that may explain observed susceptibility/protective effects. Finally, we determined the relationship of HLA-DRB1*04 population carrier frequency and latitude to GCA incidence reported in different countries.

**Results:**

In our UK data (225 cases and 1378 controls), HLA-DRB1*04 carriage was associated with GCA susceptibility (odds ratio (OR) = 2.69, *P* = 1.5×10^−11^), but HLA-DRB1*01 was protective (adjusted OR = 0.55, *P* = 0.0046). In meta-analysis combined with 14 published studies (an additional 691 cases and 4038 controls), protective effects were seen from HLA-DR2, which comprises HLA-DRB1*15 and HLA-DRB1*16 (OR = 0.65, *P* = 8.2×10^−6^) and possibly from HLA-DRB1*01 (OR = 0.73, *P* = 0.037). GCA incidence (*n* = 17 countries) was associated with population HLA-DRB1*04 allele frequency (*P* = 0.008; adjusted R^2^ = 0.51 on univariable analysis, adjusted R^2^ = 0.62 after also including latitude); latitude also made an independent contribution.

**Conclusions:**

We confirm that HLA-DRB1*04 is a GCA susceptibility allele. The susceptibility data are best explained by amino acid risk residues V, H, and H at positions 11, 13, and 33, contrary to previous suggestions of amino acids in the second hypervariable region. Worldwide, GCA incidence was independently associated both with population frequency of HLA-DRB1*04 and with latitude itself. We conclude that variation in population HLA-DRB1*04 frequency may partly explain variations in GCA incidence and that HLA-DRB1*04 may warrant investigation as a potential prognostic or predictive biomarker.

**Electronic supplementary material:**

The online version of this article (doi:10.1186/s13075-015-0692-4) contains supplementary material, which is available to authorized users.

## Introduction

Genetic association studies are a well-established method for investigating genetic contributions to disease. In rheumatoid arthritis (RA) [1] and small-vessel vasculitis [2], genetically distinct subsets have been identified that have different associations with the major histocompatibility complex (MHC) region that encodes the *HLA-DRB1* alleles. Comparison of *HLA-DRB1* associations with RA in different ethnic groups helped to support the original “shared epitope” hypothesis of RA susceptibility [3] based on an amino acid risk motif at positions 67–74 in the third hypervariable region (HVR3) of the class II MHC molecule, encoded by *HLA-DRB1.* The group of RA “shared epitope” alleles now includes HLA-DRB1*01:01, HLA-DRB1*04:01, HLA-DRB1*04:04, HLA-DRB1*04:08, and HLA-DRB1*10:01; other alleles provide weaker protective effects, additional to the risk effects of the “shared epitope” [4]. Recently, it has been demonstrated that amino acid residues 11 and 13 in the first hypervariable region (HVR1) of class II MHC display the strongest associations with RA susceptibility [5].

Giant cell arteritis (GCA) incidence is highest in populations with Scandinavian ancestry [6–8] and this has led to suggestions that this might be due to genetic factors [9, 10]. Susceptibility to GCA has been reported to be associated with carriage of HLA-DRB1*04, but not all studies have shown an association and there are conflicting data as to whether there is an association with specific HLA-DRB1*04 alleles [11]. Under-representation of HLA-DRB1*01 in GCA patients from Rochester, Minnesota, led to a suggestion that the risk of GCA may be due to a DRYF motif at positions 28–31 in the second hypervariable region (HVR2) of MHC class II [12], but a Spanish study failed to replicate this finding [13]. To date, however, formal meta-analysis has not yet been performed to determine the major susceptibility and protective *HLA-DRB1* alleles. The relative contribution of genetic and environmental factors as an explanation for geographical differences in GCA incidence also remains disputed [6, 7]. When genetic diversity within Europe is subjected to principal component analysis, the MHC is one of several genetic regions that are strongly associated with a component that runs along a north-south gradient from Norway/Sweden to Spain [14]. We therefore hypothesised that variations in the frequency of *HLA-DRB1* GCA susceptibility alleles may partly explain geographical variations in GCA incidence.

Here, we report new GCA susceptibility data, combine these with the published data using meta-analysis, and propose a new hypothesis regarding a possible amino acid GCA 11-13-33 risk motif in HVR1 and HVR2 of class II MHC. This hypothesis fits the observed data better than previously proposed models.

## Methods

### Patients

The UK GCA Consortium was designed to support genetic association studies. Investigators, all experienced rheumatologists, recruited cases with a firm clinical diagnosis of GCA, based on all available information. Recruitment was retrospective. A positive temporal artery biopsy was not required as it was not always undertaken in classic presentations or could not be performed within an optimal time window or both. In some centres, the erythrocyte sedimentation rate was unavailable and so fulfilment of the 1990 American College of Rheumatology (ACR) criteria [15], which should not be used for clinical diagnosis of GCA [16], was not a requirement for inclusion. Clinical data on a subset of this cohort have already been published [17]. In this analysis, we included all patients who agreed to give a blood sample for genetic studies up to 2012 and where a sample was available. Written informed consent was provided by all patients, and the study was approved by the York Research Ethics Committee (reference 05/Q1108/28).

### DNA extraction and genotyping

DNA was extracted from peripheral blood. *HLA-DRB1* genotyping was performed by either single-stranded oligonucleotide polymerisation [18] or allele-specific polymerase chain reactions (standard primer sequences (HLA DRBplus Typing Kit, Amersham Biosciences, now part of GE Healthcare, Little Chalfont, UK), except for the forward primer of HLA-DRB1*10 which was redesigned as 5'-GCG GTT GCT GGA AAG ACG CG-3'). Direct sequencing was also performed to enable four-digit genotyping of HLA-DRB1*04 subtypes [18] because of previous reports of a HLA-DRB1*04 association of GCA at the two-digit level. The HaplotypeViewer program was developed to facilitate rapid four-digit genotyping from sequence electropherograms and is freely available [19].

### Analysis of genotyping data

Control data from the UK Rheumatoid Arthritis Genetics (UKRAG) Consortium were used for this analysis. Initial logistic regression analyses were undertaken by assuming additive genetic models to estimate the effect of each potential susceptibility/protective allele. Adjustments for genetic effects already proposed in the literature (HLA-DRB1*04) were also performed.

### Meta-analysis of giant cell arteritis susceptibility data

To identify case-control studies of *HLA-DRB1* association with GCA susceptibility, a literature search was conducted in PubMed, without language restriction, by using the terms “HLA” and “(giant cell arteritis) OR (temporal arteritis)”. Reference lists of studies identified were also scanned. Publications were included if they provided sufficient detail on cases and controls to perform a meta-analysis. Where there were multiple publications with overlapping datasets, the report with the most complete dataset was chosen. Meta-analysis of the published summary carrier frequency data was performed (i.e., assuming a dominant mode of inheritance) because allele frequency and individual-level patient data were mostly unavailable from the authors of the studies. A random-effects model was used; the overall estimate was calculated by using as weights 1/(*v*_i_ + τ), where *v*_i_ is the variance of the estimated effect from the *i*th study and τ is the estimated between-study variance [20].

### Worldwide giant cell arteritis incidence in relation to HLA-DRB1*04 population carrier frequencies

To identify reports of the incidence of GCA in different countries, a second literature search in PubMed was conducted with combinations of the medical subject heading terms “giant cell arteritis”, “temporal arteritis”, and “epidemiology”. Hand-searching was also performed in the reference lists of retrieved articles, review articles, and textbooks. Studies were included if they were available in full-text and included an estimate of the annual incidence, time period of the study, method of case definition, population studied, and geographical location of the study. Where necessary, the incidence figure was recalculated as number of new cases per 100,000 of the over-50 population per year. Studies that appeared to report duplicate or overlapping populations were excluded. Studies completing recruitment before 1980 were excluded in case of time trends in the incidence of GCA and because the quality of the reporting was generally lower for the older studies. Where more than one report existed for a single country (unless in ethnically distinct populations), the one with a later average period of recruitment was preferred. Where a single report included two separate sub-studies (regions or time periods), a weighted mean of the two sub-studies was used to arrive at an overall incidence figure.

We then sought data on ethnically matched *HLA-DRB1* population allele frequencies at the two- and four-digit levels for each geographical region identified in the second literature search. We considered HLA-DRB1*04 alleles and those identified as being potential susceptibility/protective alleles in our own UK dataset. Methods have been reported elsewhere [21]; briefly, we first consulted the Allele Frequency Net Database [22] and then, if necessary, Ovid Medline and Embase. Carrier frequencies for control populations were converted to estimated allele frequencies by using the Hardy-Weinberg equation. Finally, the following predetermined rule was used to generate an estimate of population *HLA-DRB1* allele frequencies: reports with over 500 (four-digit typing) or 1000 (two-digit typing) controls were identified and a weighted mean calculated. In the absence of large studies, studies with more than 100 (four-digit typing) or more than 200 (two-digit typing) were identified and a weighted mean calculated. Determination of geographical latitude and linear regression analysis were performed as previously described [21].

### Development of amino acid risk motif model

After determination of susceptibility and protective *HLA-DRB1* alleles, amino acid residues in HVR1, HVR2, and HVR3 were obtained from the IMGT (International ImMunoGeneTics Information System) database [23], accessed 31 January 2012). For samples with only two-digit typing, we estimated amino acid residues on the basis of geographically relevant population frequencies of the four-digit subtypes from the Allele Frequency Net Database [22], assigning a probability to residues when they varied within the four-digit subtypes (only necessary at positions 28, 32, 37, 67, 70, 71, and 74). For each of these, the expected misclassification rate when assigned by using population frequencies is less than 1 %, apart from positions 67 (2 %) and 71 (3 %). For each polymorphic position, samples were assigned a dosage (i.e., the expected number of copies) for each residue. Logistic regression was then used to test for association at each position separately, and degrees of freedom were equal to one less than the number of distinct residues. For the most significant positions, forward stepwise regression was used to identify the residues associated with disease risk at that position.

We used population *HLA-DRB1* frequencies for inferring amino acid residues in both cases and controls. Under the null hypothesis of no association, the frequencies would be the same, and any bias introduced by using population frequencies for cases would be toward the null. Using *HLA-DRB1* four-digit frequencies that have been observed in patients with GCA to infer the amino acid residues in the GCA cases could lead to a biased analysis with inflated false-positive rate.

Results that reach a nominal significance level of 0.05 are highlighted. For the exploratory hypotheses, these should be interpreted in the light of multiple testing. Analyses were performed in SPSS 15 (IBM Corporation, Armonk, NY, USA) and Stata SE (StataCorp LP, College Station, TX, USA).

## Results

### Patients

Two hundred twenty-five patients with GCA from 7 UK centres consented to analysis of genetic material for this study (125 from Leeds hospitals, including 38 from Otley; 33 from Harrogate; 23 from Southend; 17 from York; 16 from Dewsbury; 10 from Pontefract; and one from Ipswich). Their demographics and disease characteristics, including fulfilment of 1990 ACR criteria, are shown in Table 1. Of the 183 temporal artery biopsies performed, 140 (77 %) were positive. Patients were all European Caucasian.

**Table 1:** Patient characteristics

Patient characteristic	Value (% of those with data available)	Number with data available
Female	161 (72 %)	225
Median age at onset of GCA (range), years	72 (50–94)	207
Biopsy-positive	140 (77 %)	183 (number of cases biopsied)
Median erythrocyte sedimentation rate at onset of GCA (range), mm/h	70 (5–150)	100
Median plasma viscosity at onset of GCA (range), mPa s	1.94 (1.53–2.65)	120
Median C-reactive protein at onset of GCA (range), mg/l	62.5 (<5–344)	154
Either ACR 1990 criteria for GCA fulfilled or biopsy-positive	207 (92 %)	225
Headache	184 (90 %)	204
Abnormal temporal artery as defined by ACR 1990 criteria	140 (67 %)	204
Jaw claudication	119 (57 %)	210
Visual or neurological features	116 (56 %)	207

Owing to the retrospective recruitment of cases, contemporaneously recorded data on features of giant cell arteritis (GCA) at presentation were not always recorded in the medical notes. For this reason, in 24 cases, fulfilment of American College of Rheumatology (ACR) criteria could not be documented. Six of these were biopsy-proven. In some recruiting centres, plasma viscosity or C-reactive protein was measured instead of erythrocyte sedimentation rate

### Analysis of genotyping data

Allele frequencies in cases and 1378 UKRAG controls are shown in Table 2 with per-allele odds ratios with and without adjustment for HLA-DRB1*04, the previously proposed susceptibility allele. Initial analysis was performed at the two-digit level. Four-digit analysis was also performed for the common *04 subtypes, but statistical analysis was not performed on the rarer *04 subtypes.

**Table 2:** Allele frequencies and per-allele odds ratios in giant cell arteritis cases and controls

*HLA-DRB1* allele	Allele frequency in controls, N (%)	Allele frequency in cases, N (%)	Allele frequency in biopsy-positive cases, N (%)	Unadjusted OR (95 % CI)	*P* value	OR adjusted for *04 (95 % CI)	*P* value
*01	341 (12.4)	27 (6.0)	12 (4.3)	0.46 (0.31, 0.69)	1.6×10^−4^	0.55 (0.37, 0.83)	0.0046
*03	438 (15.9)	75 (16.7)	41 (14.5)	1.06 (0.81, 1.39)	0.68	1.31 (0.99, 1.74)	0.061
*04	503 (18.3)	138 (30.7)	89 (31.6)	2.03 (1.61, 2.56)	1.7×10^−9^	-	-
*04:01	304 (11.0)	83 (18.4)	54 (19.1)	1.87 (1.42, 2.46)	8.2×10^−6^	-	-
*04:02	10 (0.4)	2 (0.4)	1 (0.4)	1.29 (0.64, 2.59)*	0.48*	-	-
*04:03	38 (1.4)	8 (1.8)	7 (2.5)				
*04:04/04:08	151 (5.5)	45 (10.0)	27 (9.6)	1.86 (1.32, 2.63)	3.7×10^−4^	-	-
*07	393 (14.3)	57 (12.7)	34 (12.1)	0.88 (0.65, 1.17)	0.37	1.03 (0.76, 1.39)	0.85
*08	78 (2.8)	8 (1.8)	4 (1.4)	0.62 (0.30, 1.29)	0.20	0.75 (0.36, 1.57)	0.45
*09	30 (1.1)	5 (1.1)	5 (1.8)	1.02 (0.39, 2.66)	0.97	1.19 (0.45, 3.12)	0.73
*10	21 (0.8)	0 (0.0)	0 (0.0)	-	-	-	
*11	179 (6.5)	32 (7.1)	20 (7.1)	1.09 (0.75, 1.59)	0.64	1.25 (0.86, 1.83)	0.25
*12	49 (1.8)	10 (2.2)	6 (2.1)	1.24 (0.64, 2.42)	0.53	1.60 (0.81, 3.14)	0.18
*13	222 (8.1)	41 (9.1)	32 (11.3)	1.14 (0.81, 1.62)	0.45	1.35 (0.95, 1.93)	0.098
*14	60 (2.2)	10 (2.2)	5 (1.7)	1.02 (0.52, 2.00)	0.95	1.29 (0.65, 2.56)	0.46
*15	421 (15.3)	45 (10.0)	32 (11.3)	0.63 (0.46, 0.87)	0.0047	0.75 (0.54, 1.04)	0.085
*16	21 (0.8)	2 (0.4)	2 (0.7)	0.58 (0.13, 2.49)	0.46	0.67 (0.16, 2.92)	0.60

*N* number of copies of allele seen in 1378 controls or 225 cases, *OR* odds ratio, *CI* confidence interval

*denotes where HLA-DRB1*04:02 and *04:03 categories were combined because of low numbers

In 225 patients with GCA and 1378 controls in the novel UK cohort, a susceptibility effect of HLA-DRB1*04 carriage was confirmed (odds ratio (OR) 2.69, 95 % confidence interval (CI) 2.02 to 3.58, *P* = 1.5×10^−11^) (Table 3). Possible protective effects from HLA-DRB1*01 and HLA-DRB1*15 were noted, but only HLA-DRB1*01 retained significance after adjusting for HLA-DRB1*04 (Table 2). The data were consistent with a dominant effect of HLA-DRB1*04 (OR for one copy 2.78, 95 % CI 2.07 to 3.72, OR for two copies 1.94, 95 % CI 0.95 to 3.96, compared with no copies).

**Table 3:** Meta-analysis of *HLA-DRB1* giant cell arteritis associations in the literature

*HLA-DRB1* allele(s)	UK GCA Cohort		Published data only				Combination of data in this report with published data			
	OR (95 % CI) for carriage of allele	*P* value	Number of studies	OR (95 % CI) for carriage of allele	Meta-analysis *P* value	Heterogeneity I^2^ statistic (%)	Number of studies	OR (95 % CI) for carriage of allele	Meta-analysis *P* value	Heterogeneity I^2^ statistic (%)
*01	0.46 (0.30, 0.70)	0.00033	14	0.78 (0.57, 1.06)	0.11	39.4	15	0.73 (0.54, 0.98)	0.037	48.1
DR2 (*15 and *16)	0.53 (0.37, 0.76)	0.00050	13	0.71 (0.57, 0.88)	0.0019	0	14	0.65 (0.54, 0.79)	8.2×10^−6^	0
*03	1.12 (0.83, 1.52)	0.47	14	1.02 (0.81, 1.28)	0.87	10.0	15	1.05 (0.88, 1.27)	0.58	4.2
*04	2.69 (2.02, 3.58)	1.5×10^−11^	15	2.45 (2.06, 2.92)	9.2×10^−24^	0	16	2.51 (2.16, 2.92)	1.1×10^−33^	0
*05 (*11 and *12)	1.21 (0.83, 1.75)	0.32	9	0.85 (0.65, 1.12)	0.24	0	10	0.96 (0.77, 1.20)	0.72	0
*06 (*13 and *14)	1.11 (0.78, 1.57)	0.56	10	0.69 (0.48, 0.99)	0.042	27.1	11	0.76 (0.55, 1.06)	0.11	40.6
*07	0.92 (0.66, 1.28)	0.63	14	1.14 (0.81, 1.60)	0.45	65.0	15	1.12 (0.83, 1.50)	0.47	63.4
*11	1.18 (0.78, 1.79)	0.43	6	0.90 (0.65, 1.26)	0.55	6.9	7	1.00 (0.77, 1.30)	0.98	5.1
*13	1.15 (0.79, 1.68)	0.47	5	0.68 (0.47, 1.00)	0.050	0	6	0.89 (0.68, 1.16)	0.39	0

*GCA* giant cell arteritis, *OR* odds ratio, *CI* confidence interval

Insufficient data were present for HLA-DRB1*08, *09, *10, *12, *14, *15 and *16 to perform meta-analysis. Published studies included in this meta-analysis for each allele were HLA-DRB1*01/*03/*07 [13, 36–48], HLA-DRB1*02 [13, 36–46, 48], HLA-DRB1*05 [13, 36–39, 41–43, 46], HLA-DRB1*06 [13, 36–39, 41–44, 46], HLA-DRB1*11 [13, 40, 44, 45, 47, 48], and HLA-DRB1*13 [13, 40, 45, 47, 48]

The effect sizes for HLA-DRB1*04 carriage were similar when restricting analyses to biopsy-positive GCA cases (OR = 2.83, 1.99 to 4.03, *P* = 7.7×10^−9^); the number of biopsy-negative GCA cases was too small for a separate analysis.

### Meta-analysis of giant cell arteritis susceptibility data

Meta-analysis of previously published data from 14 studies (691 cases, 4038 controls; Table 3) gave ORs of 2.45 (*P* = 9.2×10^−24^), 0.78 (*P* = 0.11), and 0.71 (*P* = 0.0019) for HLA-DRB1*04, *01, and *02, respectively (*02 is now reclassified as *15 and *16). There was more heterogeneity noted for some alleles than for others; the HLA-DRB1*04 meta-analysis had an I^2^ statistic of 0 % whereas the meta-analysis for HLA-DRB1*01 had an I^2^ statistic of 39.4 %. When our UK data was included, the meta-analysis still demonstrated protective effects for HLA-DRB1*01 and HLA-DRB1*02 (*P* = 0.037 and *P* = 8.2×10^−6^, respectively) (Table 3). In the six published articles with information on ethnicity, cases and controls were always stated to be either “white” or “Caucasian”. Therefore, subgroup meta-analysis by ethnic group was not possible.

### Worldwide giant cell arteritis incidence in relation to HLA-DRB1*04 population carrier frequencies

Reliable population *HLA-DRB1* data were not always available, notably for small, native tribes of Alaska and Saskatoon, where extrapolation from small and physically or genetically (or both) isolated communities was felt to be unwarranted. Table 4 summarises the GCA incidence articles included, together with the estimated population allele frequencies and the number of individuals on which these estimates are based. Substantial clinical heterogeneity was identified in the GCA incidence studies, including variations in the methods used to identify GCA cases and confirm GCA diagnosis.

**Table 4:** Incidence of giant cell arteritis and population *HLA-DRB1* allele frequencies in different countries

GCA incidence study					*HLA-DRB1* allele frequency and references (if multiple references given, the allele frequency is the weighted mean)				
City/region, country, reference	Dates, study design	GCA criteria	GCA incidence, per 100,000 per year in over-50s	Latitude (degrees N)	*04	*01	*15	*04:01	*04:04
W Nyland, Finland [49]	1987-88, prospective	Biopsy, clinical	26.2	61.5	0.147 (n = 1157 [50])	0.183 (n = 1157 [50])	0.144 (n = 1157 [50])	0.080 (n = 1157 [50])	0.037 (n = 1157 [50])
Goteborg, Sweden [51]	1976-95, retrospective	Biopsy, clinical	22.2	57.7	0.195 (n = 1347 [22], 1366 [52], 1209 [53], 1191 [54])	0.105 (n = 1347 [22], 1366 [52], 1209 [53], 1191 [54])	0.159 (n = 1347 [22], 1366 [52], 1209 [53], 1191 [54])	0.137 (n = 934 [55], 1763 [56], 1191 [54])	0.057 (n = 934 [55], 1763 [56], 1191 [54])
Reggio Emilia, Italy [57]	1980-88, retrospective	Biopsy	6.9	44.7	0.072 (n = 57,345 [58], 4080 [22], 2054 [22], 1992 [59])	0.089 (n = 57,345 [58], 4080 [22], 2054 [22], 1992 [59])	0.068 (n = 57,345 [58], 2054 [22], 1992 [59])	0.017 (n = 57,345 [58])	0.012 (n = 57345 [58])
Lugo, NW Spain [60]	1981-98, retrospective	Biopsy	10.2	43.0	0.129 (n = 1940 [61], 1088 [62])	0.103 (n = 1940 [61], 1088 [62])	0.104 (n = 1940 [61], 1088 [62])	0.053 (n = 145 [22])	0.0207 (n = 145 [22])
Denmark [63]	1982-94, prospective	Biopsy	20.4	56.5	0.180 (n = 562 [64])	0.101 (n = 562 [64])	0.157 (n = 562 [64])	0.176 (n = 55 [22])	0.001 (n = 55 [22])
Iceland [65]	1984-90, retrospective	ACR 1990 criteria	27.0	64.7	0.170 (n = 172 [66])	0.040 (n = 172 [66])	0.020 (n = 172 [66])	No data	No data
Alesund and Bodo, Norway [67]	1992-6, retrospective, hospital	Biopsy/ACR 1990 criteria	32.4	64.9	0.225 (n = 576 [22], 630 [68], 368 [69], 898 [55])	0.117 (n = 576 [22], 630 [68], 898 [55])	0.153 (n = 576 [22], 630 [68], 368 [69], 898 [55])	0.117 (n = 898 [55])	0.067 (n = 898 [55])
Schleswig Holstein, North Germany [70]	1998-2002, prospective, population	Chapel Hill consensus criteria	3.2	54.2	0.140 (n = 11,407 [22], 8862 [22], 4251 [71])	0.111 (n = 11,407 [22], 8862 [22], 4251 [71])	0.141 (n = 11,407 [22], 8862 [22], 4251 [71])	0.081 (n = 8862 [22])	0.024 (n = 8862 [22])
Vilnius, Lithuania [72]	1990-9, prospective, hospital	ACR 1990 criteria	0.7	54.7	0.078 (n = 134 [73])	0.111 (n = 134 [73])	No data	No data	No data
Jerusalem [74]	1980-2004, retrospective	Biopsy + ACR 1990 criteria	11.3	31.8	0.170 (n = 23000 [22])	0.083 (n = 23,000 [22])	0.070 (n = 23,000 [22])	0.019 (n = 132 [75])	0.015 (n = 132 [75])
Sephardic Jews, Israel [76]	1980-91, retrospective	Biopsy	10.2	31.8	0.112 (n = 293 [77])	0.038 (n = 293 [77])	0.087 (n = 293 [77])	0.000 (n = 293 [77])	0.009 (n = 293 [77])
UK [28]	1990-2001, prospective	Clinical	22.0	51.5	0.204 (n = 39,979 [22], 1238 [78])	0.110 (n = 39,979 [22], 1238 [78])	0.143 (n = 39,979 [22], 1238 [78])	0.116 (n = 298 [22])	0.047 (n = 298 [22])
Erdirne, Turkey [79]	2003-9, retrospective	Clinical	1.1	41.7	0.128 (n = 250 [22], 253 [80], 250 [81])	0.047 (n = 250 [22], 253 [80], 250 [81])	0.082 (n = 250 [22], 253 [80], 250 [81])	0.014 (n = 110 [82], 250 [81])	0.019 (n = 110 [82], 250 [81])
Olmsted County, MN, USA [83]	2000-2004, prospective, population	ACR 1990	18.9	44.0	0.156 (n = 339 [22])	0.097 (n = 339 [22])	0.124 (n = 339 [22])	0.091 (n = 339 [22])	0.040 (n = 339 [22])
White, Memphis, TN, USA [84]	1971-1980, retrospective	Biopsy/clinical	2.2	35.2	0.156 (n = 61,655 [22])	0.110 (n = 61,655 [22])	0.133 (n = 61,655 [22])	0.091 (n = 61,655 [22])	0.031 (n = 61,655 [22])
Black, Memphis, TN, USA [84]	1971-1980, retrospective	Biopsy/clinical	0.4	35.2	0.060 (n = 34 [85])	No data	0.210 (n = 34 [85])	No data	No data
Europeans, Saskatoon, Canada [86]	1998-2003, prospective	Biopsy	9.3	52.1	0.178 (n = 415 [87])	0.092 (n = 415 [87])	0.147 (n = 415 [87])	0.097 (n = 216 [88])	0.051 (n = 216 [88])

*ACR* American College of Rheumatology

Giant cell arteritis (GCA) incidence in Finland was taken as weighted mean of the two sub-studies reported in the article cited. Allele frequencies are the weighted mean of the allele frequencies reported in the studies cited (see Methods for details of how these were selected). For brevity, where the data were taken from the allelefrequencies.net website, the main citation for the website is given [22]; searching allelefrequencies.net on the country, sample size, and human leukocyte antigen (HLA) allele in question will give the allele frequencies used in this table

**Fig. 1:**
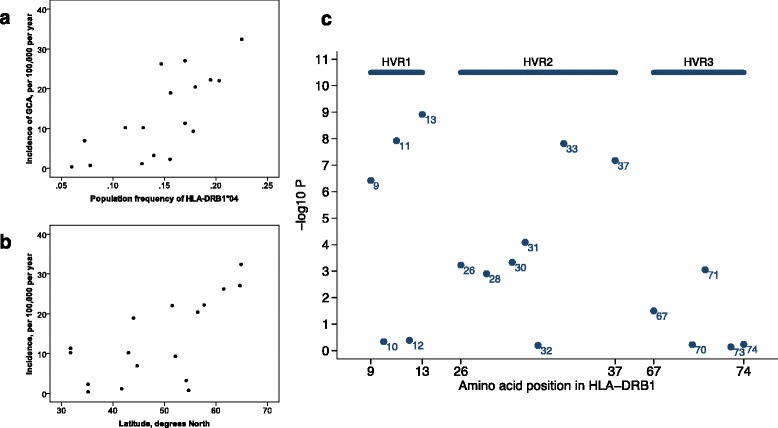
Giant cell arteritis (GCA) incidence in relation to population *HLA-DRB1* allele frequencies (**a**) and to latitude (**b**). Significance levels from a global test of difference in distribution of amino acid frequencies between cases and controls at specific positions (**c**)

At the two-digit level (Table 4 and Fig. 1a and b), 17 studies were included in the analysis of worldwide GCA incidence in relation to population *HLA-DRB1* allele frequencies. In view of our meta-analysis findings, we extracted data for HLA-DRB1*04, HLA-DRB1*01, and HLA-DRB1*15 population frequencies (Table 4)*.* The majority of these were from Europe and the Mediterranean area. Within this small dataset, HLA-DRB1*15 was more common in the general population at more northerly latitudes (*r* = 0.52, *P* = 0.038) whereas no significant association with latitude was seen for population HLA-DRB1*04 or HLA-DRB1*01 (*r* = 0.47, *P* = 0.057; *r* = 0.39, *P* = 0.133).

Predictors of GCA incidence in univariable analyses were HLA-DRB1*04 population allele frequency (*P* = 0.001, adjusted R^2^ = 0.51) and latitude (*P* = 0.004, adjusted R^2^ = 0.40), whereas HLA-DRB1*15 was non-significant. In multivariable analysis, both were significant predictors and each made independent contributions to the explanatory power of the model (HLA-DRB1*04, *P* = 0.008; latitude, *P* = 0.036; adjusted R^2^ of the model = 0.62). HLA-DRB1*15 made no additional contribution to the explanatory power of the model.

### Development of model with 11-13-33 amino acid risk motif

Tests for association showed that the most significant position was at 13 (*P* = 1.2×10^−9^), followed by 11, 33, 37, and 9 (Fig. 1c). At position 13, the most significant residue was H (OR = 2.11, 95 % CI 1.61 to 2.77, *P* = 5.5×10^−8^, equivalent to H at 33 and also to the 04 allele). However, stepwise regression found additional contributions from residues S (OR = 1.38, 95 % CI 1.07 to 1.77, *P* = 0.014) and F (OR = 0.66, 95 % CI 0.44 to 0.99, *P* = 0.038) (Table 5). Similarly, multiple residues were found at positions 11 and 37. There is very strong linkage disequilibrium in this region, and so many of these residues at different positions almost always occur together, as illustrated in Additional file 1. For example, we did not have the power to distinguish between the risk effects of V, H, and H at positions 11, 13, and 33, respectively, since the *10 allele, which differs at residue 13, is very rare. The previously proposed DYF motif (positions 28, 30, and 31) in HVR2 (OR = 1.54, 95 % CI 1.21 to 1.96, *P* = 0.00038) did not explain the observed data as well as simple HLA-DRB1*04 carriage. Similarly, variation in amino acid residues within HVR3 is unlikely to explain the observed GCA susceptibility data (Fig. 1c), especially since the other alleles comprising the “RA shared epitope” were not over-represented in GCA.

**Table 5:** Results of tests for association of amino acid at the most significant positions: 9, 11, 13, 33, and 37

Position in *HLA-DRB1*	Amino acid residue	Frequency		Univariable		Stepwise model (entry *P* = 0.05) applied separately at each position	
		Cases	Controls	OR	*P* value	OR	*P* value
9	E	0.698	0.565	1.78	7.3×10^−8^		
	K	0.011	0.011	1.03	0.96		
	W	0.291	0.424	0.56	5.8×10^−8^	0.56	5.85×10^−8^
11	D	0.011	0.011	1.03	0.96		
	G	0.127	0.142	0.88	0.38		
	L	0.060	0.123	0.46	3.6×10^−5^	0.64	0.040
	P	0.104	0.159	0.63	0.0024		
	S	0.391	0.371	1.09	0.41	1.37	0.015
	V	0.307	0.194	1.86	1.4×10^−7^	2.06	2.8×10^−7^
13	F	0.071	0.141	0.48	1.7×10^−5^	0.66	0.038
	G	0.040	0.045	0.87	0.60		
	H	0.307	0.187	1.96	1.5×10^−8^	2.11	5.5×10^−8^
	R	0.104	0.159	0.63	0.0024		
	S	0.351	0.325	1.12	0.28	1.38	0.014
	Y	0.127	0.142	0.88	0.38		
33	H	0.307	0.187	1.96	1.5×10^−8^	1.96	1.527×10^−8^
	N	0.693	0.813	0.51	1.5×10^−8^		
37	F	0.147	0.161	0.89	0.39		
	L	0.022	0.017	1.27	0.49		
	N	0.256	0.219	1.09	0.46		
	S	0.164	0.282	0.51	8.0×10^−8^	0.59	0.00012
	Y	0.402	0.276	1.63	3.2×10^−6^	1.37	0.0055

Forward stepwise regression was used to determine whether there are independent effects of more than one amino acid residue at the same position. Only amino acids that were retained by the stepwise regression model are shown in the last two columns. Odds ratio (OR) of less than 1.0 indicates a protective effect, whereas OR of more than 1.0 indicates a susceptibility effect. Note the high level of linkage disequilibrium at this locus; Additional file 1 shows the amino acid residues within the three hypervariable regions of *HLA-DRB1*

## Discussion

In this study, which includes both new UK data and the first formal meta-analysis of published data on *HLA-DRB1* associations of GCA, we not only confirm a strong association of GCA with HLA-DRB1*04 allele carriage, including within our own UK data, but also identify possible protective effects of HLA-DRB1*01 and HLA-DRB1*15, supported by the meta-analysis of previous studies. We were able to impute amino acid residues quite reliably from published allele frequencies, enabling us to analyse amino acid residues even though four-digit typing was not available for every *HLA-DRB1* allele. Based on this, it was the amino acid residues 11, 13, and 33 in the first and second hypervariable regions that best explained the observed HLA-DRB1 susceptibility and protective effects, rather than the previously proposed DRYF amino acid motif in the second hypervariable region [12]. We also observed that some non-HLA-DRB1*04 amino acid residues had additional effects (individual amino acid residues that were retained by a multivariable regression model for each separate amino acid position are shown in the last two columns in Table 5), suggesting additional genetic complexities that we did not have the power to investigate in depth. We then systematically extracted data on population prevalence of the identified susceptibility and protective *HLA-DRB1* alleles and compared this with reports of GCA incidence in different countries. We found a significant and independent relationship of GCA incidence both with HLA-DRB1*04 and with latitude. Conversely, HLA-DRB1*15 was, if anything, protective and did not contribute to incidence of GCA in the geo-epidemiological study.

Strengths of this work include the presentation of the first UK *HLA* data in GCA, its presentation in the context of the international literature, the first meta-analysis of HLA-DRB1*04 GCA susceptibility studies, and the novel approach combining a traditional genetic association study with a geo-epidemiology approach. Using logistic regression for the UK, we could control for already-known *HLA-DRB1* susceptibility effects in the per-allele analysis, which also has not been performed in other datasets, which mostly reported only carrier frequencies not allele frequencies. Based on this, we were able to suggest *HLA-DRB1* amino acid residues that best fit the observed susceptibility/protective allele effects. This is the first synthesis of the literature on reported GCA incidence in relation to population *HLA-DRB1* allele frequency and geographical latitude.

Our analysis is based on certain assumptions. Firstly, because many clinicians in the UK do not always request temporal artery biopsy except in cases of diagnostic doubt [24], we had prespecified in the analysis that GCA would be defined clinically rather than limiting inclusion to biopsy-positive cases only. The clinically diagnosed patients, however, had to be firmly diagnosed by an experienced consultant, and there had to be unequivocal clinical features and no alternative explanation for the symptoms after follow-up. Temporal artery biopsy is not 100 % sensitive for GCA; possible reasons for false-negative biopsies in our cohort included delays in obtaining biopsies resulting in resolution of inflammation, suboptimal biopsy length, and biopsy reporting based on the classic pathologic criteria, which may be overly stringent [25]; sometimes the temporal artery is spared in patients with GCA, particularly in those with predominant disease of the aorta and its proximal branches [26]. We conducted a sub-analysis of the biopsy-positive subset and found no difference in the observed effect size for HLA-DRB1*04 association compared with the whole group; with such a small number of biopsy-negative cases, no meaningful statement can be made about the effect size in that group. Our meta-analysis showed that the effect size in the cohort overall was also comparable to that observed in previous reports, some of which included only biopsy-positive cases. If not all the cases truly had GCA, this would have reduced the power of the study (“diluted out” the genetic association) but would have been highly unlikely to introduce artefactual genetic associations because the differential diagnosis of GCA is so wide. Similar pragmatic approaches to case definition for genetics studies, accepting a small, finite rate of misclassification in order to maximise recruitment, have been successfully used in other genetic association studies [27]. We also did not have the power to study whether there are differences in the effect size between regions of the UK, but regional variations in the incidence of diagnosed GCA have been described [28]; it remains unclear how far this is influenced by regional variations in population *HLA-DRB1* frequency [29].

In regard to the *HLA-DRB1* typing, it is recognised that *HLA-DRB1* represents only a small part of the whole MHC and also that not having complete sequence-based four-digit typing may have resulted in some important information being missed. This study focused on *HLA-DRB1* and we did not set out to analyse variation elsewhere in the MHC [30]. However, our finding that non-HLA-DRB1*04 residues also contributed significantly to GCA susceptibility/protection (Table 5) suggests that other alleles may also be involved. The MHC is a complex locus with extensive linkage disequilibrium, and an MHC-wide analysis requires larger datasets and specialised analysis methods. A concurrent international, collaborative large-scale genetic analysis of GCA (including samples from this study), using a different genotyping platform (Immunochip) with more extensive coverage of the HLA region [31], shows evidence of wider involvement of the MHC region while confirming the strong association with DRB1*04. Lastly, the literature reviews and meta-analyses are limited by the small number of studies in the literature, many of which were published some years ago, with corresponding variations in case ascertainment and in genotyping assays. Larger datasets using modern genotyping and statistical analysis methods will reveal further GCA susceptibility alleles within the whole *HLA* locus and allow their pattern of linkage disequilibrium to be analysed.

The *P* values reported here should be considered in the light of multiple testing, but owing to the *a priori* suggestion of HLA-DRB1*04 association and lack of consensus as to how to adjust for multiple testing at a multi-allelic locus where the different alleles are not independent of each other, we did not consider a Bonferroni correction to be appropriate here. Nevertheless, model over-fitting is a possibility, and it is essential that our findings be replicated in an independent dataset.

## Conclusions

In summary, we report a novel approach to studying genetic influences of disease by combining traditional genetic association studies with geo-epidemiology methods that capitalise on publicly available data. Our new UK data and a synthesis of the published literature suggest that HLA-DRB1*04 might explain part of the observed geographical variation in GCA incidence. This is consistent with an autoimmune aetiology for GCA [32]. However, we found additional variation in susceptibility (Table 5) and incidence (Fig. 1a, b) that is not fully explained by HLA-DRB1*04 and is likely to relate to additional, unknown genetic and environmental factors. Previous studies of GCA have also demonstrated an association between HLA-DRB1*04 and visual loss [33] and also with glucocorticoid resistance [34]. Of interest, in Japan (where HLA-DRB1*04 population frequency is low), large-vessel vasculitis (Takayasu arteritis) is relatively more common than GCA. Takayasu arteritis was associated with alleles containing the 11-13-33 V-H-H motif (HLA-DRB1*0405) in a Turkish population but was not associated with another allele also containing V-H-H (HLA-DRB1*0401) in a European-American population; HLA-DRB1*1502, which was associated with Takayasu arteritis in both populations [35], does not contain the V-H-H motif. Very few patients in our dataset had large-vessel imaging, but genetic characterisation of the subset of GCA patients who have large-vessel involvement or temporal artery sparing or both [26] would be of interest in future studies. From a clinical perspective, further study of well-phenotyped cohorts is required to determine whether HLA-DRB1*04 may serve as a biomarker of pathophysiologically relevant phenotypic disease subsets in order to develop better risk stratification, prediction of response to glucocorticoids, and ultimately targeted therapies.

## Additional files

Additional file 1:
**Appendices I and II (Membership of consortia).** Description of contents: Names and institutional affiliations of members of the UK GCA Consortium (Appendix I) and the UKRAG Consortium (Appendix II). Additional file 2:
**Amino acids at hypervariable regions (HVR) in susceptibility, protective, and neutral GCA alleles.** Table showing the amino acid residues in the hypervariable regions of *HLA-DRB1,* for each of the *HLA-DRB1* alleles. Shaded columns denote the proposed 11-13-33 GCA risk motif.

## Abbreviations

ACR: American College of Rheumatology
CI: Confidence interval
GCA: Giant cell arteritis
HLA: Human leukocyte antigen
HVR: Hypervariable region
MHC: Major histocompatibility complex
OR: Odds ratio
RA: Rheumatoid arthritis
UKRAG: UK Rheumatoid Arthritis Genetics
