# Supplementary material for: Association of HLA-DRB1 amino acid residues with giant cell arteritis: genetic association study, meta-analysis and geo-epidemiological investigation
Source: Arthritis Res Ther. 2015 Jul 30;17(1):195. doi: 10.1186/s13075-015-0692-4 (PMC4520081; doi:10.1186/s13075-015-0692-4)
Supplement: Additional file 1: — Appendices I and II (Membership of consortia). Description of contents: Names and institutional affiliations of members of the UK GCA Consortium (Appendix I) and the UKRAG Consortium (Appendix II). [file 13075_2015_692_MOESM1_ESM.docx]

Additional File 1: Amino acids at hypervariable regions (HVR) in susceptibility, protective, and neutral GCA alleles

| *HLA-DRB1* allele | HVR1 9-13 | | | | | HVR2 26-37 | | | | | | | HVR3 67-74 | | | | | |
| --- | --- | --- | --- | --- | --- | --- | --- | --- | --- | --- | --- | --- | --- | --- | --- | --- | --- | --- |
|  | 9 | 10 | 11 | 12 | 13 | 26 | 28 | 30 | 31 | 32 | 33 | 37 | 67 | 70 | 71 | 72 | 73 | 74 |
| ***04** | **E** | **Q** | **V** | **K** | **H** | **F** | **D** | **Y** | **F** | **Y** | **H** | **Y** | **L** | **Q*** | **K*** | **R** | **A** | **A*** |
| **01* | *W* | *Q* | *L* | *K* | *F* | *L* | *E* | *C* | *I* | *Y* | *N* | *S* | *L* | *Q** | *R** | *R* | *A* | *A* |
| **15* | *W* | *Q* | *P* | *K* | *R* | *F* | *D* | *Y** | *F* | *Y* | *N* | *S* | *I** | *Q* | *A* | *R* | *A* | *A* |
| *03 | E | Y | S | T | S | Y* | D* | Y | F | H | N | N | L | Q | K | R | G | R |
| *07 | W | Q | G | K | Y | F | E | L | F | Y | N | F | I | D | R | R | G | Q |
| *08 | E | Y | S | T | G | F | D | Y | F | Y | N | Y | F* | D | R | R | A | L |
| *09 | K | Q | D | K | F | Y | H | G | I | Y | N | N | F* | R* | R | R | A | E |
| *10 | E | E | V | K | F | L | E | R | V | H | N | Y | L | R | R | R | A | A |
| *11 | E | Y | S | T | S | F | D | Y | F | Y | N | Y | F* | D | R* | R | A | A |
| *12 | E | Y | S | T | G | L | E | H | F | H | N | L | I* | D | R | R | A | A |
| *13 | E | Y | S | T | S | F | D | Y | F | H* | N | N* | I | D | E* | R | A | A |
| *14 | E | Y | S | T | S* | F | D* | Y | F | H | N | F* | L | R* | R | R | A | E* |
| *16 | W | Q | P | K | R | F | D | Y | F | Y | N | S | F* | D | R | R | A | A* |

Susceptibility alleles are in bold and possible protective alleles are in italics. Alleles where no susceptibility or protective association has been suggested are in ordinary type. An asterisk denotes that there are alternative amino acids for some subtypes at the 4-digit level (first four variants at 4-digit level were checked). Shaded columns denote the proposed 11-13-33 GCA risk motif. Positions 4 and 14 are not shown because the only allele where there is a different amino acid at this position is *07, which was not shown to have a significant risk or protective effect in our data.
