# Supplementary material for: Association of HLA-DRB1 amino acid residues with giant cell arteritis: genetic association study, meta-analysis and geo-epidemiological investigation
Source: Arthritis Res Ther. 2015 Jul 30;17(1):195. doi: 10.1186/s13075-015-0692-4 (PMC4520081; doi:10.1186/s13075-015-0692-4)
Supplement: Additional file 2: — Amino acids at hypervariable regions (HVR) in susceptibility, protective, and neutral GCA alleles. Table showing the amino acid residues in the hypervariable regions of HLA-DRB1, for each of the HLA-DRB1 alleles. Shaded columns denote the proposed 11-13-33 GCA risk motif. [file 13075_2015_692_MOESM2_ESM.doc]

Additional File 2: Appendices I and II (Membership of consortia)

**Appendix I: Membership of UK GCA Consortium (at the time these samples were collected)**

Sarah L Mackie, University of Leeds and Leeds Teaching Hospitals NHS Trust, Leeds, UK

Ann W Morgan, University of Leeds and Leeds Teaching Hospitals NHS Trust, Leeds, UK

Colin T Pease, Leeds Teaching Hospitals NHS Trust, Leeds, UK

Bhaskar Dasgupta, Southend University Hospital, Southend, UK

Jane Hollywood, Southend University Hospital, Southend, UK

Andrew Gough, Harrogate and District Foundation NHS Trust, Harrogate, UK

Michael Green, York Hospitals Foundation NHS Trust, York, UK

Lesley Hordon, Dewsbury and District Hospital, Mid Yorkshire Hospitals NHS Trust, Dewsbury, UK

Stephen Jarrett, Pinderfields Hospital, Mid Yorkshire Hospitals NHS Trust, Pinderfields, UK

Shouma Dutta, Mid Yorkshire Hospitals NHS Trust, Dewsbury, Pinderfields, UK

Richard Watts, Ipwsich Hospitals NHS Trust, Ipswich, UK and Norwick Medical School University of East Anglia, Norwich, UK

**Appendix II: Membership of UKRAG Consortium**

Stephen Eyre, Anne Hinks, Laura J. Gibbons, John Bowes, Edward Flynn, Paul Martin, Wendy Thomson, Anne Barton, Jane Worthington (University of Manchester, Manchester, UK)

Stephen G. Martin, James I. Robinson, Ann W. Morgan, Paul Emery (University of Leeds, Leeds, UK)

Anthony G. Wilson (University of Sheffield, Sheffield, UK)

Sophia Steer (Kings College Hospital National Health Service Foundation Trust, London, UK)

Lynne Hocking, David M. Reid (University of Aberdeen, Aberdeen, UK)

Pille Harrison, Paul Wordsworth (University of Oxford, Oxford, UK)
